# Multiple arterial emboli secondary to left ventricular thrombus in a 35-year-old obese male

**DOI:** 10.4103/0975-3583.74264

**Published:** 2010

**Authors:** Andrew Chetwood, Alison Sanders, Martin Saweirs, Ankur Thapar, Alun H. Davies

**Affiliations:** *Department of Vascular Surgery, Charing Cross Hospital, London, UK*; 1 *Department of Emergency Medicine, Charing Cross Hospital, London, UK*

**Keywords:** Embolization, left ventricular thrombus, myocardial infarction, obesity

## Abstract

The very unusual case of a 35-year-old obese male patient with a left ventricular (LV) thrombus secondary to a silent myocardial infarction and resultant shower emboli to multiple arterial sites is described. His presentation with acute limb ischemia led to arterial imaging and the identification of the underlying cardiac pathology in addition to splenic and bilateral renal infarcts. He was also found to suffer from previously undiagnosed hypertension. He underwent femoral embolectomy and multiple arterial revascularization attempts but required bilateral above knee amputations and a prolonged intensive care unit stay. This rare and extreme example of a LV thrombus in a young male emphasizes the potential sequellae of the condition. Furthermore, with the increasing incidence of obesity this case demonstrates the importance of considering undiagnosed cardiovascular risk factors when assessing obese patients.

## INTRODUCTION

The very unusual case of multiple arterial emboli from a left ventricular (LV) thrombus following a silent myocardial infarction (MI) in a 35 year old obese male is presented. Such a severe example reminds health professionals of the potential sequellae of this recognized complication of MI[1] and to be alert to the increased risk and atypical presentation of cardiovascular disease in obese patients.

## CASE REPORT

A 35-year-old obese (BMI>40) male was transferred to the emergency department (ED) at our institution critically unwell with an acutely ischemic right lower limb. He was tachypnoeic, tachycardic and had a Glasgow Coma Score of 11 with oxygen saturations of 78% on air. On examination his right lower limb was cold and pulseless. Arterial blood-gas sampling revealed a metabolic acidosis (pH 7.18, base excess-16.9). A collateral history from his family revealed he was an ex-smoker and apart from obesity did not have any known cardiac risk factors. However, he had been experiencing bilateral loin pain for the previous 3 days and had been receiving antibiotics for a presumed pyelonephritis.

**Figure 1:**
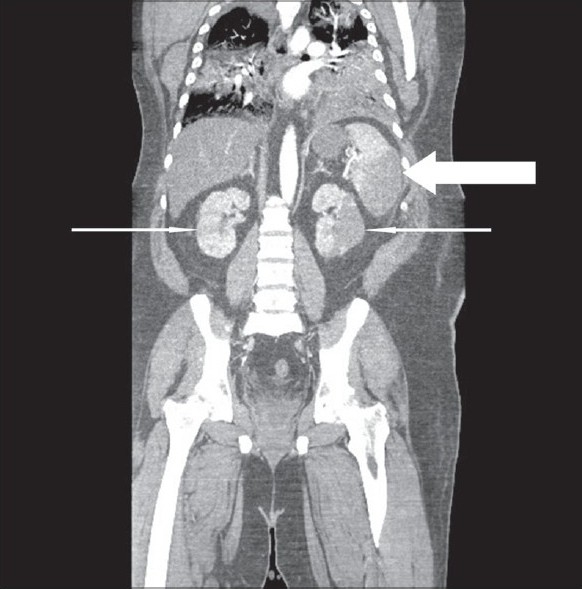
Coronal CTA image showing areas of low attenuation representing infarction within the spleen (thick arrow) and bilateral kidneys (thin arrows).

**Figure 2:**
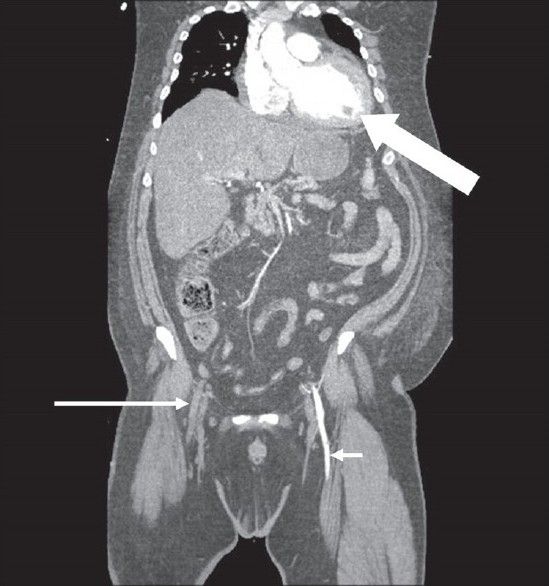
Coronal CTA image showing an occluded right external iliac/femoral artery (long arrow) when compared with the left side (short arrow). The left ventricular thrombus is visible (thick arrow)

An ultrasound performed in the ED showed a left ventricular (LV) thrombus on pericardial windows. Following stabilization and intubation, computed tomography angiography (CTA) showed occluded right external and left internal iliac arteries, an acute splenic infarct, bilateral renal infarcts and a significant filling defect in the left ventricle [Figures 1 and 2]. Intravenous heparin was commenced and he was taken to theatre where a right external iliac embolectomy and four compartment fasciotomies were performed.

**Figure 3:**
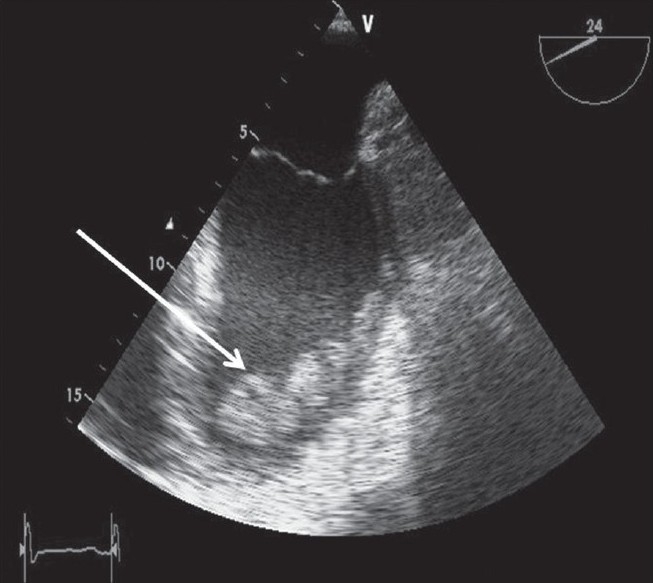
Trans-oesophageal ECHO image demonstrating thrombus within the left ventricle

Echocardiography demonstrated severe LV dysfunction (LV ejection fraction of 15%) and dilatation (LV diameter of 7 cm in systole) with an extensive 4 cm thrombus within the LV [Figure 3]. Troponin I, at admission and 24 hours, was 0.99 and 1.29 µg/L, respectively, with creatinine kinase of 131 and 16,798 U/L at the same intervals. Electrocardiogram showed T-wave inversion and poor R-wave progression in the chest leads, supporting the diagnosis of anterior myocardial infarction (MI). Cardiothoracic advice was sought and it was not thought he would survive surgical thrombectomy. Despite subsequent arterial revascularization attempts he underwent bilateral above knee amputations with a prolonged intensive care unit stay. He was discharged to a local rehabilitation unit on long-term warfarin.

At 1-year follow-up he is doing well. His cardiac function has dramatically improved (LVEF of 55%; LV systolic diameter of 4 cm) with only mildly impaired LV function and complete resolution of the LV thrombus.

## DISCUSSION

The case described provides a very rare example of the complications from a LV thrombus in such a young patient. In addition to LV aneurysm and dilated cardiomyopathy, LV thrombus can complicate MI, where akinesis promotes blood stasis and thrombus formation.[1] Embolization to distal arteries is well-recognized with the cerebral circulation being the most commonly affected.[2] However, the widespread arterial embolization to multiple anatomical sites as presented in this case is very unusual.

It is likely that the splenic and renal infarcts identified on CTA were responsible for the preceding symptom of loin pain. Renal infarction presents with loin pain and microsopic hematuria and has been reported secondary to LV thrombus. The mimicking of ureteric colic demonstrates the diagnostic difficulties associated with this condition[3] and may explain the previous misdiagnosis of his symptoms as pyelonephritis.

Obese patients can have undiagnosed cardiovascular risk factors and as such may present atypically with cardiovascular disease.[4] In this case previously undiagnosed hypertension was present. Diabetes and hypercholesterolemia are additional cardiac risk factor associated with obesity. With the incidence of obesity increasing this example serves to remind health professionals to consider additional ‘hidden’ cardiovascular risk factors when assessing obese patients and that the presentation of cardiovascular disease in this patient group may be atypical.

The causes for MI in young patients differ to the primarily atherosclerotic etiology seen in older patients. These include hypercoagulable states, spontaneous coronary artery dissection, congenital coronary artery abnormalities, accelerated atherosclerosis and coronary artery spasm.[5] The exact cause of MI in this case is not clear but it is likely that his obesity and hypertension contributed to accelerated atherosclerosis.

## CONCLUSIONS

LV thrombus can embolize to multiple arterial sites with severe results. Obese patients may have hidden cardiac risk factors and physicians need to be alert to atypical presentation of cardiovascular disease in this patient group.

## CONSENT

Informed verbal and signed consent was given by the patient in this case.
